# Mesh colposacropexy in the management of anterior vaginal compartment prolapse

**DOI:** 10.25122/jml-2019-0018

**Published:** 2019

**Authors:** Dragos Marcu, Camelia Diaconu, Lucian Iorga, Ovidiu Bratu, Dan Mischianu

**Affiliations:** 1. Clinic of Urology, “Dr. Carol Davila” Central Military Emergency University Hospital”, Bucharest, Romania; 2. “Carol Davila” University of Medicine and Pharmacy, Bucharest, Romania; 3. Academy of Romanian Scientists, Bucharest, Romania

**Keywords:** genital prolapse, mesh sacrocolpopexy, sacrospinous ligament

## Abstract

Pelvic organ prolapse is a frequent female pathology, often causing a negative impact on the patient’s quality of life. The purpose of this paper is to present the results that we have achieved in 32 patients with anterior vaginal compartment prolapse, managed using the transvaginal mesh approach.

Over a period of twelve months, we have performed 32 transvaginal reconstructive procedures using a four arms polypropylene mesh. The superior arms of the mesh have been passed through the obturator foramen while the inferior arms have been passed through the sacrospinous ligament.

The surgery has lead to a significant improvement in the quality of life in this group of patients, this being assessed using self-administered questionnaires that evaluated the quality of life, the sexual function, and urinary continence. Anatomical success was achieved in 96.87% of the cases. In terms of postoperative complications, we mention one case of vaginal erosion, one case of de novo dyspareunia and three cases of pelvic discomfort. So far we have not encountered any mesh exposure cases nor prolapse recurrence.

Considering the results that we have achieved in our study, we can conclude that the transvaginal polypropylene mesh approach can prove itself to be a viable solution for the management of genital prolapse, especially if we consider the high postoperative rates of anatomical success and low rates of postoperative complications, as well as improving the patient’s quality of life. In spite of these encouraging results, the fact that in recent years FDA has emitted several warnings in terms of postoperative complications following such procedures, as well as the fact that our study was conducted on a small group of patients, limits the strength of our research, its only purpose being to present our experience for this surgical approach over a limited period of time.

## Introduction

Pelvic organ prolapse (POP) is one of the most frequent female pathologies, and it is the result of several factors that lead to the weakening of the pelvic supporting structures (pelvic floor fascias, ligaments, and muscles). These structures act as a hammock across the opening of the pelvic floor, maintaining the pelvic organs in the normal position. The structural changes that occur with aging or as a consequence of different pelvic surgeries or to radiotherapy, together with other associated pathologies or physical activities that increase the intra-abdominal pressure will eventually lead to the descent of pelvic organs through the vagina [1–4]. The prevalence of POP rises with age, and it ranges from 6% in younger patients to over 50% in female patients that are over 50 years old. In the United States, it is estimated that approximately 25% of the female population presents a degree of POP, the risk for surgery ranging between 6% and 20% and the chance for further surgery due to prolapse recurrence being estimated to be as high as 30% [1, 5].

The etiology of POP is multifactorial, childbirth being one most important factors involved in its pathogenesis. It is well known that the number of vaginal births and the increased size of the child leads to structural changes at the level of the pelvic supporting structures during the delivery process (endopelvic fascia, pelvic muscles and the nerve branches that ensure their innervation). Other factors that increase the risk of POP are: obesity, structural changes related to aging and to hormonal imbalances as a result of menopause, history of hysterectomy, surgery for genital prolapse or other pelvic surgeries, pelvic radiotherapy for different malignancies, collagen disorders, constipation and other pathologies or situations that associate with a chronic intra-abdominal increased pressure (heavy manual labor, chronic cough) [6–16]. All of these factors weaken the pelvic floor muscles and ligaments and favor the prolapse of the pelvic organs (bladder along with the urethra, uterus, rectum) resulting in a vaginal bulge. Depending on the dimensions of the vaginal bulge, the patients can present with urinary tract symptoms (urinary urgency, urinary stress incontinence, urinary frequency, incomplete bladder emptying or difficulty in urinating, recurrent urinary tract infections), constipation or fecal incontinence, pain or discomfort during sexual intercourse, pelvic pressure or heaviness, sensation of vaginal protrusion [6, 17, 18].

The patients can present either isolated anterior vaginal wall prolapse (cystocele), posterior (rectocele) or apical prolapse (enterocele or uterine prolapse) or a combination of these types of prolapses. Anterior vaginal wall prolapse is the most frequent pelvic prolapse accounting for up to 35% of all cases, followed by posterior prolapse (18%) and apical prolapse (14%) [19]. In the case of concomitant apical prolapse, surgeons should also ensure its correction during the procedures for the anterior wall prolapse in order to obtain better long term results. Often these two types of pelvic disorders co-exist, therefore a thorough pre-operatory examination should be made to establish precisely the type of pelvic prolapse and the correct therapeutical approach because the treatment of anterior vaginal wall prolapse without the management of apical prolapse often leads to poor results and early recurrence. It is estimated that the percentage of patients who may develop recurrence can range between 25% and 40% [5, 20].

For elderly female patients, sexually inactive, with significant apical prolapse, obliterative surgical procedures such as colpocleisis and colpectomy can be considered, these procedures ensuring better and long-lasting results. In younger patients, obliterative procedures are not recommended, the management in these cases consisting in reconstructive surgery using the abdominal or vaginal approaches in order to restore the normal pelvic anatomy and to increase the patient’s quality of life [5, 21].

Over the years, the transvaginal approach using polypropylene mesh kits has become more popular among surgeons of different specialties (general surgery, urology, gynecology) when facing patients with pelvic organ prolapse. The increasing popularity of these devices is related to the fact that this type of procedure is minimally invasive, offering a fast postoperative recovery and durable results that improve the patient’s quality of life, as well as a significantly reduced morbidity rate compared to the open or laparoscopic approach [22].

The purpose of this paper is to present the results that we have achieved over a follow-up period of at least 12 months in 32 patients with anterior vaginal compartment prolapse with or without apical prolapse, managed using the transvaginal mesh approach.

## Materials and Methods

Over twelve months (June 2016 – June 2017) we have performed 32 procedures for anterior vaginal wall prolapse and apical vaginal prolapse using a minimally invasive transvaginal approach with four arms monofilament polypropylene mesh. The patients presented at least stage two genital prolapse and bothersome symptoms, the degree of prolapse being established according to the International Continence Society Pelvic Organ Prolapse Quantification system. The preoperative evaluation protocol consisted of patient history, physical examination (including the cough test), bladder ultrasound to assess the post-void residual, and urinalysis. Preoperative urodynamic studies have been recommended in all cases, but only a small number of the patients have followed this recommendation. The impact of the symptoms on the patient’s quality of life has been evaluated using the following questionnaires: Urogenital Distress Inventory, Pelvic Floor Impact Questionnaire, Pelvic Organ Prolapse – Urinary Incontinence – Sexual Questionnaire and Pelvic Floor Distress Inventory. These questionnaires have been used before and also after surgery (during the postoperative follow-up at one month, 3, 6 and 12 months) to compare the results obtained before and after surgery and to evaluate the impact of this procedure on the patient’s life quality. The menopausal patients had received prior to the surgery intravaginal estrogens in order to improve the vaginal atrophy process.

As we have previously mentioned, the mesh kit consisted of a four arms monofilament polypropylene mesh, the mesh arms being wholly made of polypropylene. We did not have a preferred manufacturer, the patients being informed about the basic specifications required for a suitable mesh. Therefore, we have used mesh kits from different manufacturers, without influencing the patient’s decision in terms of mesh kit manufacturer. We believe that an essential factor that influenced the patient’s decision in terms of choosing a particular brand was related to the cost of the mesh.

Spinal anesthesia was preferred to general anesthesia, the patients being placed in the gynecological position. After a bladder catheter was placed, the anterior vaginal wall was exposed by traction of the cervix. The procedure began with the infiltration of the anterior vaginal wall with a saline solution 9% for a better dissection. A vertical midline incision of the anterior vaginal wall was made one cm below the urethral meatus towards the cervix. After dissecting the bladder from the anterior vaginal wall and afterwards continued as far as possible posterior and laterally towards the ischial spine, a helicoidal needle was introduced through a skin incision that was made in the genito-crural line at the level of the urethral meatus, below the ischiopubic ramus, punching it through the obturator membrane and externalizing it through the vaginal incision. With the help of the helicoidal needle, the superior arms of the polypropylene mesh were passed through the obturator foramen. The inferior arms of the polypropylene mesh have been passed through the sacrospinous ligament (approximately 2 cm medially from the ischial spine) with the help of the needle used in the tension-free vaginal tape (TVT) mid-urethral sling procedure, through a skin incision which was made one cm below the ischial tuberosity. The maneuver requires special attention in order to avoid the injury of the internal pudendal vessels during the passing of the helicoidal needle through the sacrospinous ligament. All four arms were gently tightened in a tension-free manner, and the central part of the mesh was placed under the bladder, covering the dissected vesicovaginal space. After suturing the anterior vaginal wall, the patients remained with the bladder catheter for 24 hours, and a vaginal pack was inserted for 48 hours (the vaginal tampon was changed the day following the surgery).

In all the cases we have practiced a mid-urethral sling procedure (the retropubic tension-free vaginal tape approach - TVT), due to the fact that some of the patients also presented stress urinary incontinence, but also to avoid the occult stress urinary incontinence that could appear after the surgical management of the genital prolapse. After removing the bladder catheter, we have performed a bladder ultrasound every four hours to assess the post-void residual volume. If the volume exceeded 150 ml, a sterile bladder intermittent catheterization was performed.

The purpose of this procedure was to achieve anatomical success, this being defined as the absence of genital prolapse during the postoperative check-ups or the presence of a stage I prolapsed, and also to alleviate the patient’s preoperative symptoms.

## Results

Out of the 32 patients included in this study, six patients (18,75%) presented stage II genital prolapse (with bothersome symptoms), 59.37% (19 patients) stage III and seven patients stage IV prolapse. The mean age was 57.8 years, and the mean number of vaginal births was 2 (varying between one and three births). Twenty-six patients were menopausal, and 21 out of 32 patients (65.62%) were sexually active. In terms of preoperative symptoms the patients presented: vaginal globus – 27 patients (84.37%), pelvic discomfort – 26 patients (81.25%), urinary symptoms (recurrent urinary tract infections, difficulties in starting the urine flow and voiding problems) – 28 patients (87.5%), associated urinary stress incontinence – 23 patients (71.86%), dyspareunia – 18 patients.

Occult stress urinary incontinence was suspected especially in patients with high-grade prolapse. All the patients have been evaluated for occult stress urinary incontinence, and this evaluation consisted of the cough test after reducing the prolapse.

The mean operative time was 60 minutes (±10 minutes), and we have not encountered any complications during surgery. Cystoscopy was performed in all the cases after inserting the mid-urethral sling using the TVT approach in order to detect possible bladder injuries that could have appeared during the retropubic insertion of TVT needle. The bladder catheter was removed the following day, and the vaginal tampon remained for 48 hours. The mean hospitalization period was three days. Sexual activity was forbidden for 8 weeks due to the risk of mesh displacement and exposure. The postoperative periodic evaluations (performed at 1 month, 3, 6 and 12 months following surgery) consisted in local examination in order to detect mesh-related complications such as vaginal erosion, mesh displacement and/or mesh exposure, a bladder ultrasonography to evaluate the impact of the mesh on the bladder’s post-void residual volume, as well as the same self-administered questionnaires that have been used before surgery. All of the 32 patients included in this study have presented themselves for the previously described periodical postoperative follow-up.

Anatomical success was defined as the absence of prolapse during the postoperative check-ups or the presence of a stage I prolapse, and it was obtained in 96.87% of the cases (31 patients). The surgery has lead to a significant improvement of the patient’s quality of life, this being validated by the self-administered questionnaires which showed improved scores compared to those obtained before surgery. After surgery, only one patient out of the initial 18 still presented dyspareunia. Regarding the postoperative complications, we have encountered one case of de novo dyspareunia, one case of superficial vaginal erosion without mesh exposure (topic estrogens have been administrated, and the results have been favorable) and 3 cases of pelvic discomfort. During the postoperative check-ups, we did not encounter any cases of mesh exposure or prolapse relapse.

## Discussion

Over the years numerous surgical techniques have been developed and used in the management of pelvic organ prolapse. Due to the success of mesh hernia repairs and the urinary stress incontinence management using mid-urethral slings, surgeons have started applying this principle in the management of pelvic organ prolapse since the mid ’90s and in 2002 the United States Food and Drug Administration (FDA) has approved the first mesh product for POP management [1, 23].

In recent years, due to numerous complaints regarding long term complications that could arise from mesh surgery, FDA has reanalyzed the safety and efficiency of mesh products, and since 2014 these products have been reclassified as high-risk medical devices [24]. Nevertheless, the numerous advantages that this type of approach presents have made it be a first line choice for numerous surgeons when managing patients with POP. Since the reclassification of mesh kits by the FDA, several articles have underlined the role of abdominal sacrocolpopexy, either open or minimally invasive using the laparoscopic or the robot-assisted approach, reporting better long term outcomes and fewer reoperation rates [25, 26]. The disadvantages of this type of procedures are that they present longer operatory times, as well as more extended postoperative recovery periods and higher costs when compared to the transvaginal approach. During the last decade, significant progress has been made in terms of reducing the complications and disadvantages of open abdominal sacrocolpopexy, due to the development of the laparoscopic approach and the robotic-assisted sacrocolpopexy. These techniques provide better recovery and less blood loss, but there are questions regarding their efficiency in terms of postoperative success and the rate of reintervention due to prolapse recurrence, especially for the cases with large anterior vaginal wall and apical prolapse [5].

In a 2013 study, Khan has reported that the rate of reintervention for anterior compartment prolapse recurrence after laparoscopic sacrocolpopexy was higher when compared to the open abdominal approach (3.4% versus 1%) [27]. Several articles have reported that the laparoscopic sacrocolpopexy offers superior results in terms of apical prolapse management and recurrence when comparing it to transvaginal mesh surgery [25, 28–30]. In a 2014 study that compared laparoscopic sacrocolpopexy with transvaginal mesh surgery, the authors have reported that after the preoperatory counseling the older patients have opted for the transvaginal approach, whereas the younger patients preferred the laparoscopic approach. The decision of the younger female patients can be explained by the fact that the laparoscopic approach presents a lower risk of complications that could affect their sexual life and because the laparoscopic approach could achieve more durable results when compared to the vaginal approach [25].

Several studies have assessed the role of advanced age in terms of complications, morbidity and mortality rates in female patients with pelvic organ prolapse who have undergone transvaginal reconstructive surgery [31–34]. A 2014 study, conducted on 225 patients with advanced POP for whom vaginal sacrospinous ligament fixation with anterior-transobturator mesh repair was performed, has compared the results in terms of objective and subjective success, as well as the incidence of complications and the morbidity and mortality rates between elderly and younger female patients. The authors have reported that the objective success rates encountered in both groups were practically similar (93% in the group with over 75 years and 92.5% in the group under 75 years). No significant differences have been found between the two groups of patients in terms of subjective success rates, morbidity, and mortality rates [31].

Often apparently continent female patients who undergo surgery for genital prolapse develop after the surgery de novo urinary incontinence, also known as occult urinary incontinence. Therefore, to avoid further surgery for the management of de novo SUI, many surgeons prefer to perform an anti-incontinence procedure, usually the TVT approach, during the surgery for the genital prolapse [20, 35]. In a randomized clinical trial conducted on 337 continent female patients who have undergone surgery for genital prolapse, Wei has evaluated the prophylactic role of concomitant mid-urethral sling surgery to reduce the incidence of de novo SUI. The authors have reported that after the three months follow-up period the rate of de novo SUI in the group of patients who did not receive a mid-urethral sling was 49% compared to 24% in the group where an anti-incontinence procedure was performed. This significant difference was also maintained at the 1-year postoperative assessment [35].

In our study, only 9 patients did not present SUI prior to surgery. During the surgery, we have also inserted a mid-urethral sling in the TVT manner (in all the cases), to correct the already existing SUI which was found in 23 cases or to avoid the postoperative occult SUI. Until now, the results have been favorable, none of the patients complaining of SUI after the surgery.

Mesh exposure is a severe complication that in the majority of cases requires reintervention to remove the exposed mesh. According to literature data, the incidence of mesh exposure varies widely from 0% to 33% [36–39]. Despite this, we have not identified any such case in our study so far.

## Conclusions

Pelvic organ prolapse is a frequent pathology, encountered especially in female patients aged over 50 years and who have given birth to more than one child. Often this pathology can have a negative impact on the patient’s life, limiting their normal daily activities due to the fact that a large number of women who suffer from pelvic organ prolapse associated symptoms develop depression and present the tendency of self-isolation.

Considering the results that we have achieved in our study, we can conclude that the transvaginal polypropylene mesh approach can prove itself to be a viable solution for the management of genital prolapse, especially if we consider the high postoperative rates of anatomical success and low rates of postoperative complications, as well as improving the patient’s quality of life. In spite of these encouraging results, the fact that in recent years the FDA has emitted several warnings in terms of postoperative complications following such procedures, as well as the fact that our study was conducted on a small group of patients, limits the strength of our study, its only purpose being to present our experience for this surgical approach over a limited period of time.

### Strengths

In terms of strengths, we mention the fact that the procedures have been performed by a single team, highly specialized in urogynecological pathology and surgery, with vast experience in similar transvaginal mesh procedures. Validated questionnaires have been used in all cases before and after the surgery to evaluate the patient’s quality of life and to assess the role of this particular approach in the management of genital prolapsed and its bothersome symptoms.

### Limitations

The major limitation of our study we consider it to be the fact that this study was conducted on a small group of patients over a limited period of time. This fact may limit its strength despite the good results that we have encountered during the postoperative follow-up. Therefore, we are aware that a much larger number of patients being is needed to achieve a meaningful statistical conclusion, but the purpose of this paper was to present our findings for this particular approach.

## Conflict of Interest

The authors confirm that there are no conflicts of interest.
